# Identification of Candidate Genes for Economically Important Carcass Cutting in Commercial Pigs through GWAS

**DOI:** 10.3390/ani13203243

**Published:** 2023-10-18

**Authors:** Fuchen Zhou, Jianping Quan, Donglin Ruan, Yibin Qiu, Rongrong Ding, Cineng Xu, Yong Ye, Gengyuan Cai, Langqing Liu, Zebin Zhang, Jie Yang, Zhenfang Wu, Enqin Zheng

**Affiliations:** 1 College of Animal Science and National Engineering Research Center for Breeding Swine Industry, South China Agricultural University, Guangzhou 510642, China; zfc17854225519@163.com (F.Z.); qjp_scau@outlook.com (J.Q.); ruandl@stu.scau.edu.cn (D.R.); 13422157044qyb@gmail.com (Y.Q.); drr_scau@foxmail.com (R.D.); cnxu@stu.scau.edu.cn (C.X.); yinhun0517@163.com (Y.Y.); cgy0415@163.com (G.C.); langqing.liu@scau.edu.cn (L.L.); zbzhang@scau.edu.cn (Z.Z.); jieyang2012@hotmail.com (J.Y.); 2 Guangdong Provincial Key Laboratory of Agro-Animal Genomics and Molecular Breeding, South China Agricultural University, Guangzhou 510642, China; 3 Guangdong Zhongxin Breeding Technology Co., Ltd., Guangzhou 510642, China; 4 Yunfu Subcenter of Guangdong Laboratory for Lingnan Modern Agriculture, Yunfu 527400, China

**Keywords:** pigs, fine segmentation, tenderloin weight, rib weight, genome-wide association studies

## Abstract

**Simple Summary:**

Tenderloin and rib weight are important components of the economic value of pig carcasses, and selling them separately after fine segmentation further enhances the economic value of the carcasses. This study represents one of the rare attempts to conduct a genome-wide analysis focused on the economic value of pig carcasses, utilizing post-slaughter carcass phenotype values and genotype data to identify genetic variation regions. Through our investigation, we have identified several promising candidate regions and genes that have the potential to contribute valuable insights for breeding strategies and marker-assisted selection in pig production.

**Abstract:**

During the process of pork production, the carcasses of pigs are divided and sold, which provides better economic benefits and market competitiveness for pork production than selling the carcass as a whole. Due to the significant cost of post-slaughter phenotypic measurement, the genetic architecture of tenderloin weight (TLNW) and rib weight (RIBW)—important components of pig carcass economic value—remain unknown. In this study, we conducted genome-wide association studies (GWAS) for TLNW and RIBW traits in a population of 431 Duroc × Landrace × Yorkshire (DLY) pigs. In our study, the most significant single nucleotide polymorphism (SNP) associated with TLNW was identified as ASGA0085853 (3.28 Mb) on Sus scrofa chromosome 12 (SSC12), while for RIBW, it was Affx-1115046258 (172.45 Mb) on SSC13. Through haplotype block analysis, we discovered a novel quantitative trait locus (QTL) associated with TLNW, spanning a 5 kb region on SSC12, and a novel RIBW-associated QTL spanning 1.42 Mb on SSC13. Furthermore, we hypothesized that three candidate genes, *TIMP2* and *EML1*, and *SMN1*, are associated with TLNW and RIBW, respectively. Our research not only addresses the knowledge gap regarding TLNW, but also serves as a valuable reference for studying RIBW. The identified SNP loci strongly associated with TLNW and RIBW may prove useful for marker-assisted selection in pig breeding programs.

## 1. Introduction

Pork is the most widely consumed meat globally and serves as a crucial protein source in human nutrition [1,2]. Due to diverse dietary preferences, pork consumption varies across regions, resulting in substantial price discrepancies for different pork cuts. Carcass segmentation enables the separate sale of distinct parts, thereby enhancing the overall market value of the carcass and providing better economic benefits and market competitiveness for pork production [3,4]. In the Chinese market, tenderloin represents the highest-priced fresh meat [5,6], and customers particularly appreciate tenderloin for its attributes such as juiciness, low fat content, and high protein content [7,8]. Ribs are important components of the pig carcass and provide a maximum reflection of the pig’s economic value. Therefore, tenderloin weight (TLNW) and rib weight (RIBW) as the quantitative traits are the key indicators of pig carcass segmentation.

However, the high costs associated with slaughter testing and the challenges in data collection have posed significant obstacles in unraveling these genetic mechanisms. Limited research has been conducted specifically focusing on post-slaughter carcass traits. Rib number, as an indirect indicator of rib weight, is influenced by a diverse array of genetic factors. Notably, the *VRTN* gene, associated with vertebral development, has undergone extensive investigation and emerged as a promising candidate gene for regulating rib number. It is localized within the quantitative trait loci (QTL) region of swine chromosome 7 [6,9,10,11,12,13]. Additionally, previous studies have shed light on other factors influencing rib growth, including the *LTBP2* gene, which has demonstrated its capability to enhance rib number in knockout mice [5]. In their study on the regulation of muscle development, Van Laere et al. [14] discovered that the *IGF2* gene plays a crucial role in muscle development in pigs. Oczkowicz et al. [15] found that the *IGF2* gene leads to a significant increase in tenderloin weight (11 ± 0.01 g). Furthermore, Burgos et al. [16] reported the capacity of the *IGF2* gene to enhance tenderloin muscle tissue in pigs. Xie et al. [13] identified the *CD96* gene as a potential candidate gene for influencing tenderloin. However, there is currently limited genetic structural analysis directly targeting post-slaughter carcass traits, and there are still many uncertainties in the genetic mechanisms underlying these traits.

In recent years, the rapid advancement and application of high-throughput sequencing technology have propelled genome-wide association studies (GWAS) as a robust strategy for identifying genetic variations associated with complex traits. This approach has found extensive use in the fields of husbandry [17,18,19] and human disease research [20,21]. Compared to previous studies employing microsatellite molecular markers [22,23], the combination of GWAS with single nucleotide polymorphism (SNP) molecular marker technology offers greater accuracy in detecting QTL intervals [24]. GWAS has also been extensively employed to investigate the genetic variation and diversity underlying economically important traits in pigs [25,26]. In previous studies [27,28], GWAS, utilizing genotype information from the GeneSeek Porcine 50K SNP Chip, has successfully identified numerous significant QTLs and candidate genes associated with important economic traits.

To further pinpoint the key loci influencing carcass segmentation traits, we performed a GWAS on the post-slaughter traits of TLNW and RIBW in a cohort of 431 Duroc × Landrace × Yorkshire (DLY) pigs. The aim of this study was to unravel the genetic architecture underlying carcass segmentation traits and facilitate the rapid development of molecular breeding in pigs.

## 2. Materials and Methods

### 2.1. Ethics Statement

The animals and experimental procedures used in this study were handled following the guidelines set forth by the Animal Care and Use Committee of the South China Agricultural University (SCAU) (Guangzhou, China). The ethics committee of SCAU approved all animal experiments. The experimental animals were not anesthetized or euthanized during this study.

### 2.2. Samples and Phenotype Data

**Figure 1:**
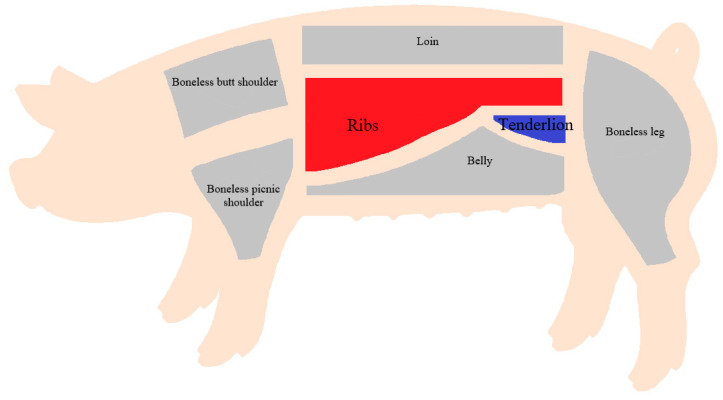
Schematic diagram of carcass cutting after pig slaughter.

In the present study, we collected 431 three-way crossbred DLY pigs from Wens Foodstuff Group Co., Ltd. (Yunfu, China). All pigs were subjected to the same growth and feeding conditions. After that, the unified slaughtering was carried out according to the standard slaughtering flow at (110 ± 5) kg body mass. Pigs fasted for 24 h before slaughter, were provided with free drinking water, and were subjected to neither beating nor driving before slaughtering, so as not to affect the meat slaughtering experiment carried out in the slaughterhouse. All pigs were divided into five batches and slaughtered in a commercial abattoir in Chifeng, Inner Mongolia. Electric shock anesthesia and heart bloodletting were used for slaughtering, and fine segmentation of each pork carcass was subsequently carried out. After fine segmentation, the weight of either the tenderloin or the ribs was recorded, as TLNW and RIBW, respectively (431 pigs for TLNW, 408 pigs for RIBW). Further details on the segmentation position are shown in Figure 1.

### 2.3. SNP Genotyping and Quality Control

The genomic DNA needed in this experiment was isolated and extracted from the ear tissue of 431 pigs using the standard phenol/chloroform method. All 431 DNA samples were subjected to DNA quality control according to light absorption ratio (A260/280 and A260/230), gel electrophoresis, and DNA concentration of 50 ng/μL. The GeneSeek pig 50 K SNP chip was used for genotyping with a total of 50,643 SNPs. The genotype quality control of the 431 DLY pigs was conducted using PLINK v1.9 software [29]. Individuals with a call rate of less than 95% and SNPs with a call rate of less than 90% and a minor allele frequency of less than 0.01 were deleted. SNPs that failed the Hardy–Weinberg equilibrium test (*p* < 10^−6^) and were unmapped or located on the sex chromosome were also removed. After QC, 4188 SNPs not located on autosome chromosomes were discarded. Moreover, we removed 211 (TLNW) and 213 (RIBW) SNPs because of missing genotype data; 11,202 SNPs due to failing the Hardy–Weinberg exact test; and 64 SNPs due to their minor allele threshold. All animals passed the QC (431 for TLNW and 408 for RIBW). Finally, 34,978 TLNW SNPs and 34,976 RIBW SNPs were retained for subsequent analysis.

### 2.4. Population Structure and Single-Locus GWAS Analysis 

Population stratification is one of the main reasons for unreliable GWAS results, as it can cause false positive results. Principal component analysis (PCA) and LD analysis were performed using the SNPs that met the QC standards to investigate the population structure. PCA was performed with GCTA software (version 1.93.2 beta) [30]. In addition, the quantile–quantile (Q–Q) plot and inflation factor (λ) were obtained using the qqman package in R software (version 4.1.2).

GEMMA software (version 0.98.5) was used to implement a Mixed Linear Model (MLM) for single-locus GWAS of TLNW and RIBW [31]. GEMMA calculated the genome correlation matrix (GRM) between individuals in each population to illustrate the population structure. The first five principal components calculated by the GCTA tool are embedded into the correlation analysis model as covariables to eliminate the mixed influence of population structure [32]. The model for testing the allelic effects of TLNW and RIBW invoked by each SNP to GEMMA is as follows:

y=Wα+Xβ+u+ε

where y represents a vector of TLNW and RIBW; W is the incidence matrix of covariates, including fixed effects of the top three eigenvectors of sex, live weight, slaughter batch, and the top five principal component from PCA analysis; α represents the vector of corresponding coefficients including the intercept; X is the vector of all marker genotypes; β specifies the corresponding effect size of the marker size; u is the vector of random effects, with $u\sim {\mathrm{MVN}}_{n}\;\left(0,\;\lambda \tau^{-1}K\right)$; ε is the vector of random residuals, with $\varepsilon \sim {\mathrm{MVN}}_{n}\;\left(0,\;\tau^{-1}In\right)$; λ signifies the ratio between two variance components; $\;\tau^{-1}$ is the variance of the residual errors; K is the GRM; I is an *n* × *n* identity matrix; and n refers to the number of pigs. In the study, Bonferroni correction was used to determine the threshold *p* values of single-locus GWAS. At a stringent genome-wide Bonferroni threshold, *p* < (0.05/N). At a more lenient threshold, *p* < (1/N) for chromosome-wide (suggestive) associations, and N means the number of SNPs [25]. Haploview v4.2 software was used to perform haplotype block analysis to estimate the LD pattern of significant SNPs in an LD block [33]. LD among SNPs were estimated as the squared correlation (r2) of alleles with a window size of 1000 kb.

The model in GCTA software, as following, is used to estimate the SNP-based heritability and the phenotypic variance explained by genome-wide SNPs (based on SNP inheritance), the proportion of phenotypic variation explained by significant SNPs:

$$y=X\beta +g+\varepsilon \;\mathrm{with}\;\mathrm{var}(y)={Ag\sigma }_{g}^{2}+{I\sigma }_{\varepsilon }^{2}$$

where *y* is the vector of tenderloin weight or ribs weight; *β* is the vector including fixed effects; *X* is an incidence matrix for *β*; *g* is the vector of the aggregate effects of all the qualified 50K SNPs for the pigs within one population; Ι is the identity matrix; *Ag* is the genomic relatedness matrix estimated by these SNPs; $\sigma_{g}^{2}$ is the additive genetic variance captured by either the genome-wide SNPs or the selected SNPs; and $\sigma_{\varepsilon }^{2}$ is the residual variance. The heritability and the phenotypic variance explained by the SNPs can be estimated using the model simply described as h^2^ = $\sigma_{g}^{2}/\sigma_{p}^{2}$*,* where $\sigma_{p}^{2}$ (total phenotypic variance) is the sum of $\sigma_{g}^{2}$ and $\sigma_{\varepsilon }^{2}$.

### 2.5. Identification of Candidate Genes and Functional Analysis

All SNPs refer to the latest version of the Sus scrofa 11.1 genome (http://ensembl.org/Sus_scrofa/Info/Index, accessed on 3 August 2023). Functional gene annotation (v105) was downloaded in GIFF3 format from the Ensembl website (http://ftp.ensembl.org/pub/release-105/gff3/sus_scrofa/, accessed on 3 August 2023). The R package BioMart (version 2.56.1) [34] efficiently retrieved functional genes. KEGG and GO analyses were conducted using KOBAS 3.0 [35] to investigate the functions of all candidate genes. Enriched terms with a significance threshold of *p* value < 0.05 were selected to further explore the genes invoked in pathway and biological processes. Subsequently, we employed REVIGO (http://revigo.irb.hr, accessed on 8 August 2023) in conjunction with the Mus musculus database to eliminate GO term redundancy (medium threshold, 0.7) and cluster the remaining terms in a 2D space [36,37]. This space was derived by applying multidimensional scaling to a matrix of GO terms with semantic similarities. The Mouse Genome Informatics website (https://www.informatics.jax.org/, accessed on 10 August 2023), GeneCards (http://www.genecards.org/, accessed on 10 August 2023), and Ensembl (www.ensembl.org/biomart/martview, accessed on 10 August 2023) were used to query gene functions.

## 3. Results

### 3.1. Phenotypic Variation and SNP Genotyping

Table 1 presents various phenotypic and genetic parameters for TLNW and RIBW traits, including animal count, mean, standard deviation, maximum, minimum, coefficient of variation (CV), and heritability. On average, TLNW and RIBW were 0.46 ± 0.08 kg and 4.51 ± 0.56 kg, respectively. The CV values for TLNW and RIBW were 17.40% and 12.42%, respectively. The distribution and visualization of the SNP dataset across chromosomes are summarized in Figure S1, Tables S1 and S2. These SNPs were roughly proportionally distributed across all 18 chromosomes of pigs, with the longest chromosome having the highest number of SNPs. Importantly, the SNP-based heritability (including standard errors) for TLNW and RIBW were 0.42 (0.11) and 0.22 (0.09), respectively.

### 3.2. Single-Locus GWAS for TLNW and RIBW

**Figure 2:**
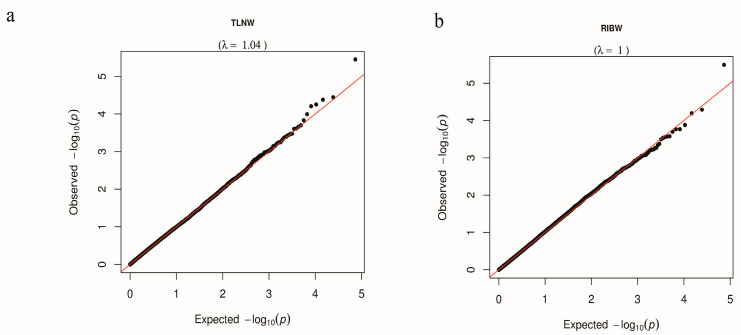
The Q–Q plots of TLNW (**a**) and RIBW (**b**) for DLY population. The Q–Q plot shows the observed versus expected −log10 *p* value. The red line represents observed values equal to expected values.

Population stratification is a significant factor contributing to the unreliability of GWAS data, as it can result in false positive findings. The quantile–quantile (Q–Q) plots serve as a valuable tool for assessing the presence of population stratification [38]. In our study, the genomic inflation factors (λ) for TLNW and RIBW GWAS were determined to be 1.04 and 1.00, respectively (Figure 2). These values suggest that the TLNW and RIBW data obtained from the DLY population in our study are not influenced by population stratification.

**Figure 3:**
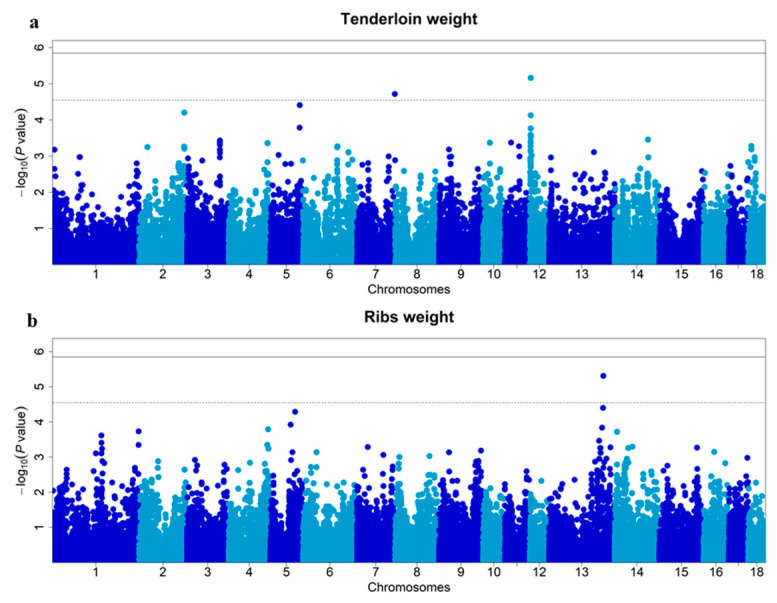
Manhattan plots of GWAS for TLNW (**a**) and RIBW (**b**) in the DLY population. In the Manhattan plots, the solid and dashed lines represent the 5% genome-wide and chromosome-wide (suggestive) Bonferroni-corrected thresholds, respectively. The x-axis represents the chromosomes, and the y-axis represents the −log10 (*p* value).

**Figure 4:**
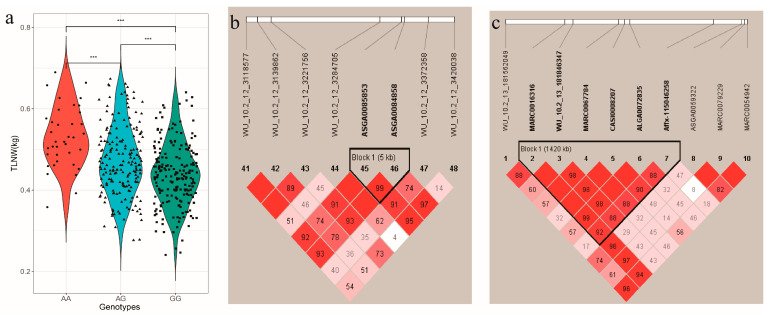
The plot (**a**) indicates the genotype effect plot of top SNP (ASGA0085853) related to TLNW in 431 DLY pigs (*** *p* < 0.01). Haplotype block for (**b**) TLNW and (**c**) RIBW in DLY pigs, respectively. Haplotype blocks are marked with triangles. Values in boxes are the linkage disequilibrium (r2) between the SNP pairs. The haplotype blocks are colored in accordance with the standard Haploview color scheme: LOD > 2 and D′ = 1, red; LOD < 2 and D′ < 1, white (LOD is the log of the likelihood odds ratio, a measure of confidence in the value of D′).

The mixed model was used to perform a single marker test, aiming to identify genetic markers associated with the TLNW and RIBW traits. The significant SNPs distinguished by single-locus GWAS for TLNW and RIBW are shown in Figure 3 and Table 2. The chromosome-wide (suggestive) Bonferroni-corrected thresholds of TLNW and RIBW were *p* < 2.86 × 10^−5^ (1/34,978) and *p* < 2.86 × 10^−5^(1/34,976), respectively. Furthermore, two suggestive SNPs (ASGA0085853 and ALGA0112188) were found to be associated with TLNW on Sus scrofa chromosome (SSC) 12 and SSC7, respectively, while one suggestive SNP (Affx-1115046258) was related to RIBW on SSC13. Moreover, Figure 3a shows that the most significant cluster is on SSC12, indicating a strong signal. The most significant SNPs for TLNW and for RIBW were ASGA0085853 and Affx-1115046258, respectively. Additionally, on SSC12, ASGA0085853 is positioned at 3.28 Mb with a minor allele frequency (MAF) of 0.306, yielding a −log10 (*p*-value) of 5.16. On SSC13, Affx-115046258 is located at 172.45 Mb with a MAF of 0.268 and a −log10 (*p*-value) of 5.31. The most significant SNPs (ASGA0085853 and Affx-1115046258) for the above characterized haplotype block an explained 4.88% and 5.19% of the phenotypic variance for TLNW and RIBW, respectively. Among these, carriers of the A allele (AA and AG) of ASGA0085853 had significantly greater loin weight than those with the GG genotype, with highly significant phenotypic differences observed among the three genotypes (Figure 4a). However, for Affx-1115046258, there were no significant differences in phenotype among the three genotypes (Figure S2).

### 3.3. Effects of the QTL for TLNW and RIBW

Haploview v4.2 [33] can visualize the linkage disequilibrium (LD) and/or linkage between significant SNPs on the same chromosome, forming block and linkage value. The QTL regions recognized by Haploview v4.2 are shown in Figure 4. The leading SNPs (ASGA0085853, Affx-115046258) were mapped to two QTL regions spanning 5 kb and 1.42 Mb, respectively. For TLNW, one QTL region was identified on SSC12, which was composed of only two SNPs located between 3,284,259 and 3,289,920 bp (Figure 4b). In addition, there is very strong linkage between ASGA0085853 (*p*-value = 6.88 × 10^−6^) and ASGA0084858 (*p*-value = 7.45 × 10^−5^). For RIBW, the QTL region on SSC13 was composed of six SNPs; the most significant, Affx-115046258 (172.45 Mb), was linked closely with the other five SNPs (MARC0016316, WU_10.2_13_181846347, MARC0067784, CASI0008207, ALGA0072835) in the QTL region (Figure 4c). As illustrated in Figure 3b and Figure 4c, CASI0008207 is the second most significant SNP in the GWAS results of the RIBW trait.

### 3.4. Candidate Genes Search and Functional Annotation

**Figure 5:**
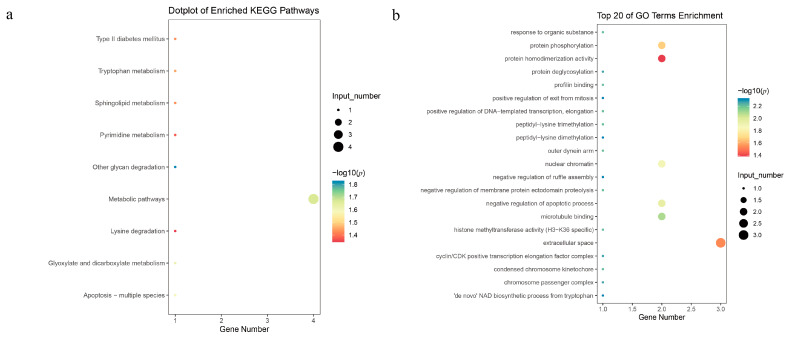
Significant KEGG pathways and GO terms associated with TLNW (*p* < 0.05). The plot (**a**) represents the KEGG pathway of the biological process for protein-coding genes within a 1 Mb region centered on the significant SNPs. The plot (**b**) shows the top 20 terms of the GO enrichment.

**Figure 6:**
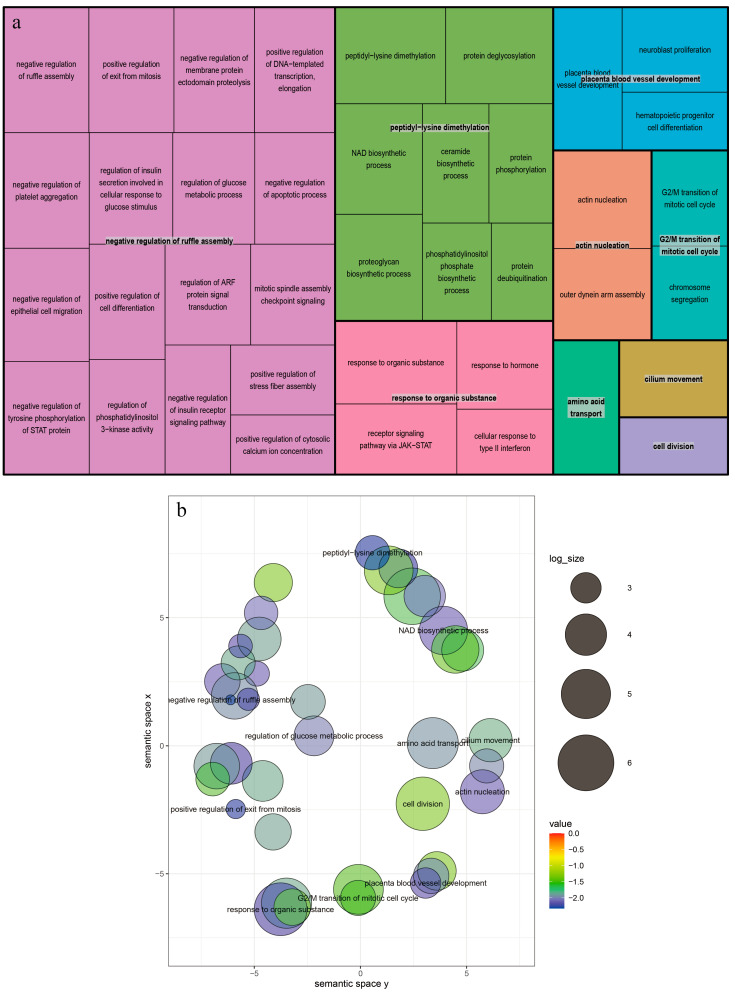
Non-redundancy GO terms of TLNW. The plot (**a**) shows the “TreeMap” view of REVIGO. Each rectangle represents a representative cluster. These representatives are combined into “superclusters,” representing loosely related terms and visualized using different colors. The size of the rectangles is adjusted to reflect the *p* value and frequency of the GO term in the Mus musculus GOA database. The plot (**b**) displays the scatter plot of representative clusters. The log size indicates the frequency of the GO term in the Mus musculus GOA database, with larger sizes indicating more common terms. The numerical value represents the −log10 (*p* value), with colors ranging from red to blue indicating increasing significance.

In the analysis of TLNW, within a range of 500 kb upstream and downstream of the significant SNPs, we annotated 11 and 13 protein-coding genes on SSC7 and SSC12, respectively (Table S3). Notably, both of the significant SNPs were located within the *TIMP2* and *EML1* genes. Our pathway enrichment analysis revealed several significantly enriched terms from the Kyoto Encyclopedia of Genes and Genomes (KEGG) and the Gene Ontology (GO) knowledgebase that are relevant to TLNW. These enriched terms include cellular division and protein translation (Figure 5a,b, Table S4). After conducting non-redundant GO analysis on all GO terms that exceeded the threshold using the REVIGO website, a total of 39 GO terms were clustered (Figure 6a,b). The most prominent signaling pathway among them is negative regulation of ruffle assembly (GO:1900028). Subsequently, we employed the GeneCards—Mouse Genome Informatics databases—and conducted an extensive literature review to explore the functional roles of the identified genes. As a result, we identified a total of four candidate genes with potential relevance to TLNW. These genes, namely *YY1*, *EML1*, *CANT1,* and *TIMP2*, exhibit promising associations with TLNW based on their known functions and previous research findings. Furthermore, we identified the *SMN1* gene as a strong candidate gene for the RIBW trait. The *SMN1* gene is homologous to the *ENSSSCG00000029127* gene and is located downstream of the leading SNP by approximately 150.8 kb.

## 4. Discussion

### 4.1. Fine Segmentation and Sale of Pig Carcasses

According to the 2021 edition of the OECD-FAO Agricultural Outlook, the global meat supply is projected to expand, reaching 374 million tons by 2030 (https://www.oecd-ilibrary.org/agriculture-and-food/oecd-fao-agriculturaloutlook_19991142, accessed on 1 August 2022). While the demand for pork is gradually rising worldwide, many nations have preferences for specific varieties of meat from pig carcasses [39]. For example, Germans consume fresh shoulder pork more frequently than any other portion [40], while Denmark is known for its high frequency consumption of liver [40]. In South Korea, fresh pork belly is an extremely popular meat, accounting for 59% of the per capita consumption of approximately 100 g of meat per day [41]. Additionally, TLN and RIB are highly favored by Chinese people due to their excellent meat quality, despite being relatively expensive compared to other parts [42]. Slaughtering and selling pig carcasses through fine segmentation can satisfy different consumers’ preferences for pork, showcase the true value of hogs, and fully explore the subsequent processes of existing animal husbandry production. Pig producers can enhance pig performance and increase economic value by utilizing genetic structure analysis of the TLNW and RIBW traits. 

### 4.2. Genetic Loci and Candidate Genes for the TLNW Trait 

The leading SNP (ASGA0085853) was annotated within the first intron of the *Metallopeptidase inhibitor 2* (*TIMP2*) gene on SSC12 through Sus scrofa 11.1 delivered from the Ensembl database. In mice, deficiency of *TIMP2* leads to increased cardiac hypertrophy and subsequent heart enlargement [43]. In cows, mRNA expression of *TIMP2* is associated with intramuscular fat content and explains 32% of the variation in intramuscular fat [44]. *TIMP2* also affects feed conversion efficiency in cattle by regulating cell growth and proliferation networks [45,46]. Based on these findings, *TIMP2* may regulate the size of cardiomyocytes by influencing the molecular pathways of cell growth and proliferation, ultimately affecting the development of the TLNW trait. On SSC12, the candidate gene *CANT1* is located 79.8 kb upstream of the leading SNP and is associated with abnormal skeletal morphology and body size in mice [47]. Additionally, *EML1*—found on SSC7, a candidate gene associated with TLNW—is located within ALGA0112188. *EML1* is associated with brain overgrowth syndrome [48] and plays a crucial role in proper retinal lamination during cellular proliferation and development [49] in humans. The potential function of *EML1* suggested by this study requires further investigation for functional validation. On SSC7, the candidate gene, *YY1*, has been shown to be involved in cell proliferation and body size in mice [50]. It is suggested that *YY1* might regulate tenderloin growth through processes related to the proliferation and development of muscle cells. These results indicate that the *TIMP2* and *EML1* genes may play an important role in TLNW and should be considered strong candidate genes for this trait. 

### 4.3. Genetic Loci and Candidate Genes for the RIBW Trait 

After conducting haplotype block analysis, we identified *ENSSSCG00000049210* and *ENSSSCG00000050907* as noncoding genes within the QTL on SSC13. However, we discovered a protein-coding gene, *ENSSSCG00000029127*, located 150.8 kb downstream of the leading SNP (Affx-115046258). Interestingly, *ENSSSCG00000029127* exhibits homology to the protein encoded by the *SMN1* gene. Our literature search revealed that the *SMN1* gene is the determinant gene for spinal muscular atrophy in humans [51], a rare hereditary neuromuscular disease caused by deletion and/or mutation of *SMN1* [52]. The *SMN1* gene has been demonstrated to be associated with physiological conditions such as abnormal muscle physiology, decreased body weight and size, and abnormal motor neuron morphology [53,54,55]. Additionally, Lorson et al. [56] reported the first cloning and identification of the porcine *SMN1* gene, showing significant sequence homology between porcine and human *SMN1* in the entire coding region. Schrank et al. [57] demonstrated that the *SMN1* gene may be involved in early embryonic death. It is possible that the *SMN1* gene may influence the development of the RIBW trait by affecting proximal muscle atrophy caused by the degeneration of spinal motoneurons in pigs. However, the specific molecular mechanism underlying this relationship requires further investigation.

## 5. Conclusions

In this study, we performed a GWAS to investigate the TLNW trait and RIBW trait in a population of 431 DLY pigs. We identified two suggestive SNPs (ASGA0085853 and ALGA0112188) associated with TLNW, and one SNP (Affx-1115046258) associated with RIBW. Furthermore, we discovered two novel QTL regions on SSC12 (5 kb) and SSC13 (1.42 Mb) that were significantly related to TLNW and RIBW, respectively. Notably, the QTL region on SSC12 represents the first association with the TLNW trait reported to date. During further analysis, we identified three major candidate genes: *TIMP2* and *EML1* for TLNW, and *SMN1* for RIBW. This research provides valuable insights for segmenting carcass molecular breeding strategies.

## Figures and Tables

**Table 1:** Summary statistics of tenderloin weight and ribs weight.

Trait	N ^3^	Mean (±SD)/kg ^4^	Min/kg ^5^	Max/kg ^6^	C.V./% ^7^	h^2^ (±SE) ^8^
TLNW ^1^	431	0.46 ± 0.08	0.24	0.69	17.40	0.42 ± 0.11
RIBW ^2^	408	4.51 ± 0.56	2.87	6.17	12.42	0.22 ± 0.09

^1^ Tenderloin weight (TLNW). ^2^ Ribs weight (RIB). ^3^ Number of animals (N). ^4^ Standard deviations (SD). ^5^ Minimum (Min). ^6^ Maximum (Max). ^7^ Coefficient of variation (C.V.). ^8^ Heritability (standard error) value (h^2^ (±SE)).

**Table 2:** Significant SNPs for TLNW and RIBW in DLY Pigs.

Trait	SNP	SSC ^1^	Position (bp)	EPV ^2^	MAF	*p*-Value	Distance ^3^	Nearest Gene
TLNW	ASGA0085853 ALGA0112188	12 7	3,284,259 120,821,692	4.88% 3.90%	0.306 0.325	6.88 × 10^−6^ 1.92 × 10^−5^	within within	*TIMP2* *EML1*
RIBW	Affx-115046258	13	172,454,121	5.19%	0.268	4.88 × 10^−6^	150.8 kb	*ENSSSCG00000029127*

^1^ Sus scrofa chromosome (SSC). ^2^ Explained phenotypic variance (EPV). ^3^ The SNP located upstream/downstream of the nearest gene (Distance).

## Data Availability

The SNP genotyping data containing variant information for the DLY pigs are not publicly available because the genotyped animals belong to commercial breeding companies, but they can be obtained from the corresponding author under reasonable requirements.

## Supplementary Materials

The following supporting information can be downloaded at: https://www.mdpi.com/article/10.3390/ani13203243/s1, Figure S1: Distribution of SNPs across Chromosomes after quality control for TLNW (a) and RIBW (b); Figure S2: The genotype effect plot of top SNP (Affx-115046258) related to RIBW in 408 DLY pigs (*** *p* < 0.01, ns *p* > 0.05); Table S1: Distributions of SNPs after QC and the average SNPs on each chromosome of Tenderloin weight traits; Table S2: Distributions of SNPs after QC and the average SNPs on each chromosome of Ribs weight traits; Table S3: The distribution of genes within a 1 Mb range around significantly associated SNPs for the TLNW trait; Table S4: This file provides the enrichment of protein-coding genes in KEGG pathway and GO terms.
