# Supplementary material for: Identification of Candidate Genes for Economically Important Carcass Cutting in Commercial Pigs through GWAS
Source: Animals (Basel). 2023 Oct 18;13(20):3243. doi: 10.3390/ani13203243 (PMC10603759; doi:10.3390/ani13203243)
Supplement: Supplementary file 1 [file animals-13-03243-s001.zip › Table S1.pdf]

Table S1. Distributions of SNPs after QC and the average SNPs on each chromosome for tenderloin weight (TLNW) traits.

SSC	SNP no.	chr. size (Mb)[1]	SNP density (SNP/Mb)
1	3425	274.33	12.48
2	2376	151.94	15.64
3	2216	132.85	16.68
4	2207	130.91	16.86
5	1688	104.53	16.15
6	2268	170.84	13.28
7	2123	121.84	17.42
8	2147	138.97	15.45
9	2347	139.51	16.82
10	1170	69.36	16.87
11	1486	79.17	18.77
12	1040	61.60	16.88
13	2736	208.33	13.13
14	2282	141.76	16.10
15	2097	140.41	14.93
16	1343	79.94	16.80
17	1093	63.49	17.22
18	934	55.98	16.68

[1]The physical size is based on Sus scrofa Build 11.1 (http://www.ensembl.org/Sus_scrofa/Info/Index)
