# Supplementary material for: Identification of Candidate Genes for Economically Important Carcass Cutting in Commercial Pigs through GWAS
Source: Animals (Basel). 2023 Oct 18;13(20):3243. doi: 10.3390/ani13203243 (PMC10603759; doi:10.3390/ani13203243)
Supplement: Supplementary file 1 [file animals-13-03243-s001.zip › Table S2.pdf]

Table S2. Distributions of SNPs after QC and the average SNPs on each chromosome for ribs weight (RIBW) traits.

SSC	SNP no.	chr. size (Mb)[1]	SNP density (SNP/Mb)
1	3424	274.33	12.48
2	2376	151.94	15.64
3	2216	132.85	16.68
4	2208	130.91	16.86
5	1688	104.53	16.15
6	2268	170.84	13.28
7	2123	121.84	17.42
8	2149	138.97	15.46
9	2347	139.51	16.82
10	1169	69.36	16.85
11	1486	79.17	18.77
12	1039	61.60	16.87
13	2734	208.33	13.12
14	2282	141.76	16.10
15	2096	140.41	14.93
16	1344	79.94	16.81
17	1093	63.49	17.22
18	934	55.98	16.68

[1]The physical size is based on Sus scrofa Build 11.1 (http://www.ensembl.org/Sus_scrofa/Info/Index)
