# Supplementary material for: Identification of Candidate Genes for Economically Important Carcass Cutting in Commercial Pigs through GWAS
Source: Animals (Basel). 2023 Oct 18;13(20):3243. doi: 10.3390/ani13203243 (PMC10603759; doi:10.3390/ani13203243)
Supplement: Supplementary file 1 [file animals-13-03243-s001.zip › Table S3.pdf]

Table S3. The distribution of genes within a 1 Mb range around significantly associated SNPs for the TLNW trait.

SNP	SSC[1]	Postion/ bp	Distance/ kb [2]	Gene
ASGA0085853	12	3,284,259	145.42	*RBFOX3*
ASGA0085853	12	3,284,259	134.92	*ENGASE*
ASGA0085853	12	3,284,259	114.18	*C1QTNF1*
ASGA0085853	12	3,284,259	79.77	*CANT1*
ASGA0085853	12	3,284,259	61.55	*LGALS3BP*
ASGA0085853	12	3,284,259	within	*TIMP2*
ASGA0085853	12	3,284,259	-24.98	*USP36*
ASGA0085853	12	3,284,259	-71.99	*CYTH1*
ASGA0085853	12	3,284,259	-198.39	*DNAH17*
ASGA0085853	12	3,284,259	-305.28	*PGS1*
ASGA0085853	12	3,284,259	-359.67	*SOCS3*
ASGA0085853	12	3,284,259	-463.02	*BIRC5*
ASGA0085853	12	3,284,259	-475.45	*AFMID*
ALGA0112188	7	120,821,692	335.01	*SETD3*
ALGA0112188	7	120,821,692	307.68	*CCNK*
ALGA0112188	7	120,821,692	224.00	*CCDC85C*
ALGA0112188	7	120,821,692	126.68	*CYP46A1*
ALGA0112188	7	120,821,692	Within	*EML1*
ALGA0112188	7	120,821,692	-89.96	*EVL*
ALGA0112188	7	120,821,692	-236.41	*DEGS2*
ALGA0112188	7	120,821,692	-310.50	*YY1*
ALGA0112188	7	120,821,692	-370.74	*SLC25A47*
ALGA0112188	7	120,821,692	-391.29	*WARS1*
ALGA0112188	7	120,821,692	-416.58	*WDR25*

[1] Sus scrofa chromosome (SSC). [2] The SNP located upstream/downstream of the gene (Distance).
