# Supplementary material for: Identification of Candidate Genes for Economically Important Carcass Cutting in Commercial Pigs through GWAS
Source: Animals (Basel). 2023 Oct 18;13(20):3243. doi: 10.3390/ani13203243 (PMC10603759; doi:10.3390/ani13203243)
Supplement: Supplementary file 1 [file animals-13-03243-s001.zip › Table S4.pdf]

Table S4. The enrichment of protein-coding genes in KEGG pathway and GO terms.

Term	Database	ID	Input number	*P*_Value
Other glycan degradation	KEGG PATHWAY	ssc00511	1	0.014797786
Metabolic pathways	KEGG PATHWAY	ssc01100	4	0.021403412
Glyoxylate and dicarboxylate metabolism	KEGG PATHWAY	ssc00630	1	0.024036051
Apoptosis - multiple species	KEGG PATHWAY	ssc04215	1	0.025567719
Tryptophan metabolism	KEGG PATHWAY	ssc00380	1	0.035467895
Type II diabetes mellitus	KEGG PATHWAY	ssc04930	1	0.03698247
Sphingolipid metabolism	KEGG PATHWAY	ssc00600	1	0.03698247
Pyrimidine metabolism	KEGG PATHWAY	ssc00240	1	0.042265666
Lysine degradation	KEGG PATHWAY	ssc00310	1	0.045272229
negative regulation of ruffle assembly	Gene Ontology	GO:1900028	1	0.004695702
'de novo' NAD biosynthetic process from tryptophan	Gene Ontology	GO:0034354	1	0.004695702
positive regulation of exit from mitosis	Gene Ontology	GO:0031536	1	0.004695702
peptidyl-lysine dimethylation	Gene Ontology	GO:0018027	1	0.004695702
chromosome passenger complex	Gene Ontology	GO:0032133	1	0.005476275
protein deglycosylation	Gene Ontology	GO:0006517	1	0.005476275
cyclin/CDK positive transcription elongation factor complex	Gene Ontology	GO:0008024	1	0.005476275
profilin binding	Gene Ontology	GO:0005522	1	0.006256264
outer dynein arm	Gene Ontology	GO:0036157	1	0.006256264
histone methyltransferase activity (H3-K36 specific)	Gene Ontology	GO:0046975	1	0.006256264
response to organic substance	Gene Ontology	GO:0010033	1	0.006256264

peptidyl-lysine trimethylation	Gene Ontology	GO:0018023	1	0.006256264
positive regulation of DNA-templated transcription, elongation	Gene Ontology	GO:0032786	1	0.006256264
condensed chromosome kinetochore	Gene Ontology	GO:0000777	1	0.006256264
negative regulation of membrane protein ectodomain proteolysis	Gene Ontology	GO:0051045	1	0.006256264
peptidyl-lysine monomethylation	Gene Ontology	GO:0018026	1	0.007035671
placenta blood vessel development	Gene Ontology	GO:0060674	1	0.007035671
mitotic spindle midzone	Gene Ontology	GO:1990023	1	0.007035671
negative regulation of platelet aggregation	Gene Ontology	GO:0090331	1	0.007035671
cyclin-dependent protein serine/threonine kinase activator activity	Gene Ontology	GO:0061575	1	0.007035671
branching involved in labyrinthine layer morphogenesis	Gene Ontology	GO:0060670	1	0.007035671
NAD biosynthetic process	Gene Ontology	GO:0009435	1	0.007814496
ATP-dependent microtubule motor activity, minus-end-directed	Gene Ontology	GO:0008569	1	0.007814496
actin nucleation	Gene Ontology	GO:0045010	1	0.007814496
negative regulation of epithelial cell migration	Gene Ontology	GO:0010633	1	0.007814496
negative regulation of tyrosine phosphorylation of STAT protein	Gene Ontology	GO:0042532	1	0.007814496
microtubule binding	Gene Ontology	GO:0008017	2	0.008036249
1-phosphatidylinositol-3-kinase regulator activity	Gene Ontology	GO:0046935	1	0.008592738

trophoblast giant cell differentiation	Gene Ontology	GO:0060707	1	0.008592738
dynein complex	Gene Ontology	GO:0030286	1	0.0093704
protein-lysine N-methyltransferase activity	Gene Ontology	GO:0016279	1	0.0093704
histone methyltransferase activity (H3-K4 specific)	Gene Ontology	GO:0042800	1	0.0093704
regulation of insulin secretion involved in cellular response to glucose stimulus	Gene Ontology	GO:0061178	1	0.0093704
proteoglycan biosynthetic process	Gene Ontology	GO:0030166	1	0.0093704
regulation of glucose metabolic process	Gene Ontology	GO:0010906	1	0.01014748
metalloendopeptidase inhibitor activity	Gene Ontology	GO:0008191	1	0.01014748
RNA polymerase II CTD heptapeptide repeat kinase activity	Gene Ontology	GO:0008353	1	0.01014748
chromosome, centromeric region	Gene Ontology	GO:0000775	1	0.01014748
outer dynein arm assembly	Gene Ontology	GO:0036158	1	0.01092398
dynein light intermediate chain binding	Gene Ontology	GO:0051959	1	0.01092398
neuroblast proliferation	Gene Ontology	GO:0007405	1	0.01092398
negative regulation of apoptotic process	Gene Ontology	GO:0043066	2	0.011229508
amino acid transport	Gene Ontology	GO:0006865	1	0.0116999
phosphatidylinositol 3-kinase complex	Gene Ontology	GO:0005942	1	0.01247524
regulation of phosphatidylinositol 3-kinase activity	Gene Ontology	GO:0043551	1	0.01247524
PcG protein complex	Gene Ontology	GO:0031519	1	0.01247524
positive regulation of cell differentiation	Gene Ontology	GO:0045597	1	0.01247524
ARF guanyl-nucleotide exchange factor activity	Gene Ontology	GO:0005086	1	0.013250001

regulation of ARF protein signal transduction	Gene Ontology	GO:0032012	1	0.013250001
dynein intermediate chain binding	Gene Ontology	GO:0045505	1	0.013250001
receptor signaling pathway via JAK-STAT	Gene Ontology	GO:0007259	1	0.013250001
mitotic spindle pole	Gene Ontology	GO:0097431	1	0.013250001
nuclear chromatin	Gene Ontology	GO:0000790	2	0.013395221
negative regulation of insulin receptor signaling pathway	Gene Ontology	GO:0046627	1	0.014024183
response to hormone	Gene Ontology	GO:0009725	1	0.014024183
actin polymerization or depolymerization	Gene Ontology	GO:0008154	1	0.014024183
positive regulation of cyclin-dependent protein serine/threonine kinase activity	Gene Ontology	GO:0045737	1	0.014024183
mitotic spindle assembly checkpoint	Gene Ontology	GO:0007094	1	0.014024183
cysteine-type endopeptidase inhibitor activity involved in apoptotic process	Gene Ontology	GO:0043027	1	0.014797786
cilium movement	Gene Ontology	GO:0003341	1	0.014797786
response to cytokine	Gene Ontology	GO:0034097	1	0.015570811
cyclin-dependent protein serine/threonine kinase activity	Gene Ontology	GO:0004693	1	0.017115129
ceramide biosynthetic process	Gene Ontology	GO:0046513	1	0.018657139
mitotic spindle assembly	Gene Ontology	GO:0090307	1	0.020196845
positive regulation of mitotic cell cycle	Gene Ontology	GO:0045931	1	0.020965835
protein phosphorylation	Gene Ontology	GO:0006468	2	0.021286943
phosphotyrosine residue binding	Gene Ontology	GO:0001784	1	0.021734251
spindle microtubule	Gene Ontology	GO:0005876	1	0.021734251

cyclin-dependent protein serine/threonine kinase regulator activity	Gene Ontology	GO:0016538	1	0.022502091
sperm flagellum	Gene Ontology	GO:0036126	1	0.023269358
tubulin binding	Gene Ontology	GO:0015631	1	0.025567719
positive regulation of stress fiber assembly	Gene Ontology	GO:0051496	1	0.025567719
G2/M transition of mitotic cell cycle	Gene Ontology	GO:0000086	1	0.025567719
positive regulation of actin filament polymerization	Gene Ontology	GO:0030838	1	0.026332694
chromosome segregation	Gene Ontology	GO:0007059	1	0.027097096
mitotic spindle organization	Gene Ontology	GO:0007052	1	0.027097096
mitotic cytokinesis	Gene Ontology	GO:0000281	1	0.027860928
collagen trimer	Gene Ontology	GO:0005581	1	0.027860928
cytoplasmic microtubule	Gene Ontology	GO:0005881	1	0.028624188
extracellular space	Gene Ontology	GO:0005615	3	0.029203476
scavenger receptor activity	Gene Ontology	GO:0005044	1	0.030910546
collagen binding	Gene Ontology	GO:0005518	1	0.031671525
negative regulation of cysteine-type endopeptidase activity involved in apoptotic process	Gene Ontology	GO:0043154	1	0.032431936
phosphatidylinositol phosphorylation	Gene Ontology	GO:0046854	1	0.033951051
phagocytic vesicle	Gene Ontology	GO:0045335	1	0.037738908
cysteine-type endopeptidase activity	Gene Ontology	GO:0004197	1	0.03849478
axoneme	Gene Ontology	GO:0005930	1	0.039250086
SH3 domain binding	Gene Ontology	GO:0017124	1	0.040004828
protein homodimerization activity	Gene Ontology	GO:0042803	2	0.041607835

mitotic spindle	Gene Ontology	GO:0072686	1	0.042265666
cellular response to interferon-gamma	Gene Ontology	GO:0071346	1	0.042265666
positive regulation of MAPK cascade	Gene Ontology	GO:0043410	1	0.042265666
protease binding	Gene Ontology	GO:0002020	1	0.043770073
motile cilium	Gene Ontology	GO:0031514	1	0.043770073
cell division	Gene Ontology	GO:0051301	1	0.043770073
positive regulation of cytosolic calcium ion concentration	Gene Ontology	GO:0007204	1	0.043770073
hematopoietic progenitor cell differentiation	Gene Ontology	GO:0002244	1	0.045272229
microtubule-based movement	Gene Ontology	GO:0007018	1	0.046772138
negative regulation of inflammatory response	Gene Ontology	GO:0050728	1	0.046772138
positive regulation of protein kinase B signaling	Gene Ontology	GO:0051897	1	0.046772138
protein deubiquitination	Gene Ontology	GO:0016579	1	0.048269802
