# Supplementary figures and images for: Identification of Candidate Genes for Economically Important Carcass Cutting in Commercial Pigs through GWAS
Source: Animals (Basel). 2023 Oct 18;13(20):3243. doi: 10.3390/ani13203243 (PMC10603759; doi:10.3390/ani13203243)

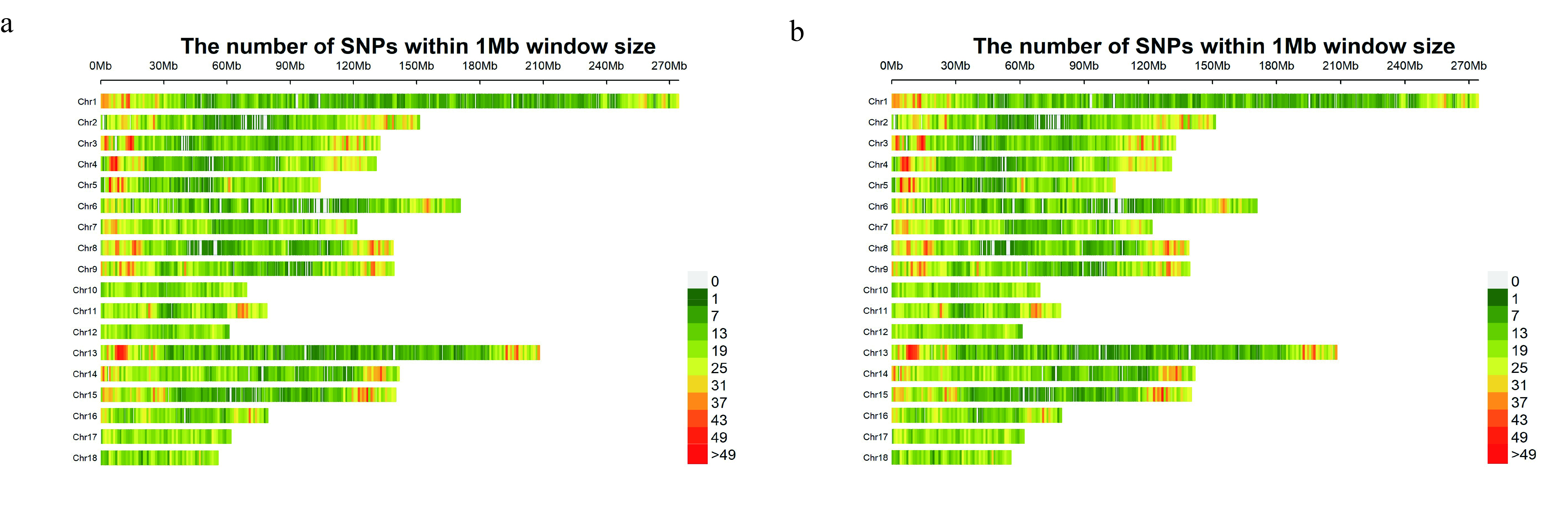

Supplement: Supplementary file 1 [file animals-13-03243-s001.zip › Figure S1.tif]

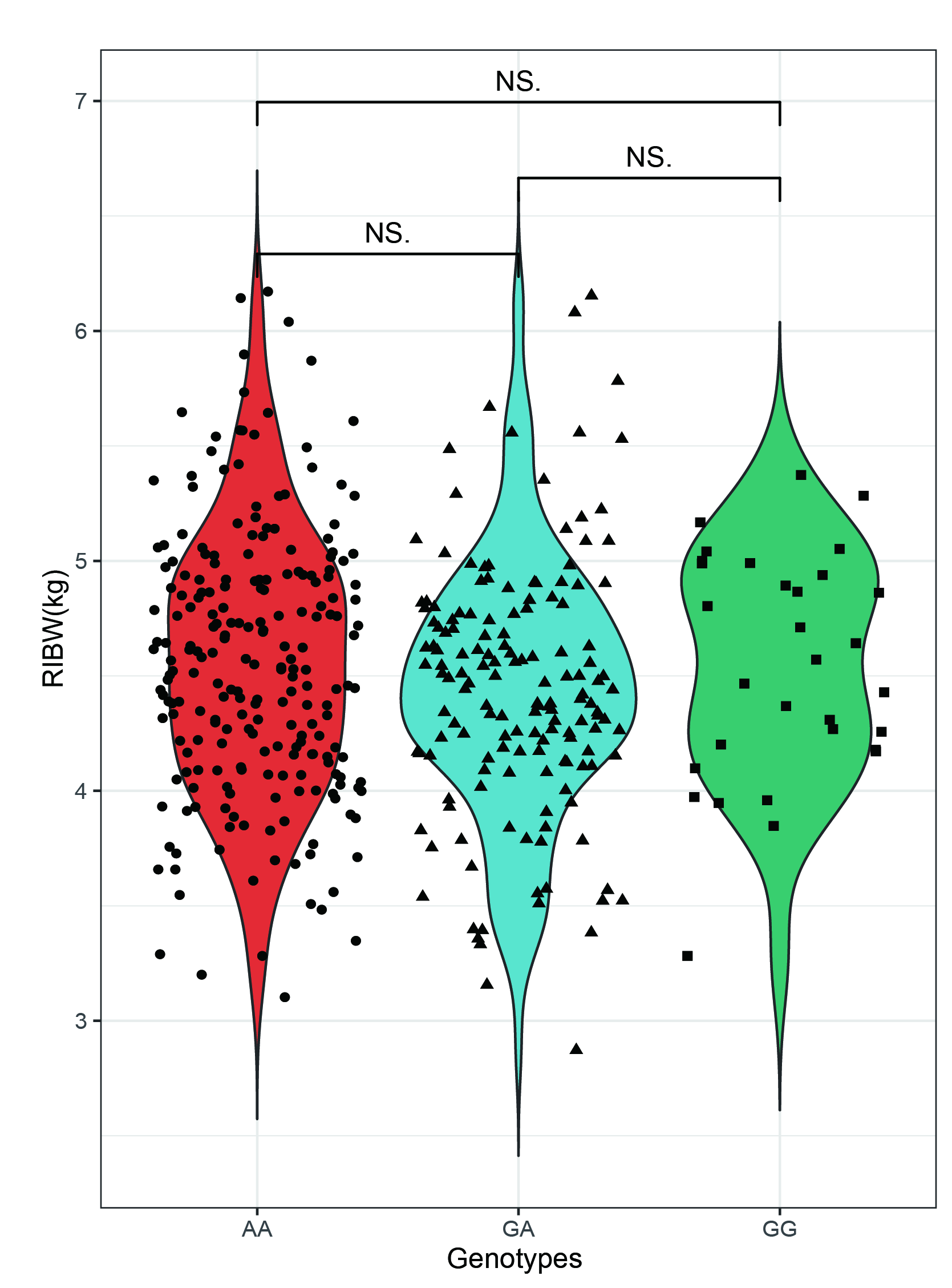

Supplement: Supplementary file 1 [file animals-13-03243-s001.zip › Figure S2.tif]
